# Formal evidence-based medicine instruction in Turkish undergraduate medical education: an initial evaluation

**DOI:** 10.1186/s12909-021-02876-5

**Published:** 2021-08-19

**Authors:** Özlem Serpil Çakmakkaya

**Affiliations:** Department of Medical Education, Istanbul University-Cerrahpaşa, Cerrahpaşa Medical Faculty, 34098 Fatih, Istanbul, Turkey

**Keywords:** Evidence-Based Medicine, Program development, “Education, Medical”, Fresno Test, Program assessment

## Abstract

**Background:**

Global and national undergraduate medical education accreditation organizations recommend the inclusion of Evidence-Based Medicine (EBM) instructions into the medical schools’ curricula. Accordingly, some Turkish medical schools have individually developed and implemented EBM training programs, but there is no data of current programs’ effectiveness and students’ learning achievements due to the lack of a validated Turkish language EBM assessment tool.

This study evaluates the effect of a newly introduced formal EBM instruction to the curriculum on students’ knowledge and skills by using the recently published Turkish adaptation of the Fresno Test.

**Methods:**

The study is an experimental investigation using pre- and post-test evaluations. A five-week EBM course was developed according to Kern’s six-step curriculum development approach. A total of 78 students from the third (*n* = 30), fourth (*n* = 19) and fifth (*n* = 29) year of medical school voluntarily consented and were enrolled into the course. Overall, the Cerrahpaşa Medical Faculty had a total of 555, 461, and 400 students enrolled in the third, fourth, and fifth year, respectively.

The program has been evaluated based on students’ learning achievements and survey responses.

**Results:**

The students’ mean pre-test Fresno Test score improved from 49.9 ± 18.2 to 118.9 ± 26.3 post-training. The Cohen’s effect size was 3.04 (95% CI, 2.6–3.5). The overall students’ satisfaction score was 8.66 ± 1.09 on a 1 to 10 scale.

**Conclusions:**

The program was effective in improving students’ knowledge and skills on EBM. We propose to offer the program as an elective course during the third year of the medical school curriculum based on all data obtained during the program evaluation.

## Background

Evidence-Based Medicine (EBM) is the conscientious explicit and judicious use of current best evidence in combination with clinical experience in making decisions about the care of individual patients [1]. Practicing EBM requires knowledge and skills. The lack of knowledge and skills is one important factor that hinders the practice of EBM [2]. Today, national and international educational organizations and boards emphasize that EBM training should be included in the medical schools’ curricula. However, no consensus has yet been reached on the most effective training methods or strategies [3]. Educational research studies are not sufficient to draw a clear conclusion, but these studies can help to suggest important principles for developing new EBM training programs [4]. Based on these suggested principles, the content of the training program should cover the basic steps of EBM, the educational methods should be interactive, student-centered, clinically integrated and the EBM training should be introduced in the pre-clinical years [4–6]. Considering these main principles, medical schools can develop a training program that is in line with their education programs’ goals and objectives, the needs of their target groups, and their resources [3, 6].

In Turkey, the national accreditation standards for undergraduate medical education recommend that medical schools should include evidence-based medical practices in their program [7]. This standard is a quality development standard, not a basic standard yet, which means that teaching EBM is currently not strictly required. Accordingly, some Turkish medical schools have individually developed and implemented EBM training programs, but other medical schools did not establish such programs. There is no data of current EBM programs’ effectiveness and students’ learning achievements due to the lack of a validated Turkish language EBM assessment tool; previous studies mostly report students’ perspectives [8, 9]. This study is the first Turkish study that reports students’ knowledge and skills in EBM before and after a newly implemented formal instruction using the recently published Turkish adaptation of the Fresno Test [10].

The aim of the study was to evaluate the effect of a new EBM training program on students’ knowledge and skills, which was developed based on current literature suggestions and our medical school needs and resources.

## Methods

This study was carried out at Istanbul University – Cerrahpaşa, Cerrahpaşa Medical Faculty (CMF), after it was approved by the CMF ethics committee. All volunteer students gave written informed consent before the pre-test and start of the course. The study was designed and conducted in accordance with the principles of the Declaration of Helsinki.

The study is a cross-sectional experimental investigation using pre- and post-test evaluations.

### Participants

CMF has two different programs based on primary teaching language, a Turkish and an English program. Students of both programs were invited to participate in the EBM training. Participants were attending the third, fourth and fifth year of medical school. Undergraduate medical education takes six years in Turkey. The newly developed EBM course was offered to all students in their clinical years except for sixth year students. Most of the students take the residency entry exam at the end of the sixth year, and preparation usually does not allow for additional voluntary coursework. The aim was to teach 25 students from each of the three classes on a voluntary basis. To account for possible dropouts, up to 30 students from each year were allowed. The announcement of the voluntary new EBM program was posted on the web page of the medical school, and an application form was provided.

Students who did not complete one of the pre- or post-tests or who were absent from two or more classes were excluded from the study. A total of 78 students from the third (*n* = 30), fourth (*n* = 19) and fifth (*n* = 29) year of medical school voluntarily consented and were enrolled into the course. Overall, the CMF Turkish Program had a total of 447, 367, and 300 students enrolled in the third, fourth, and fifth year, respectively. The English Program had 108, 94, and 100 students in the same order.

### Educators

Lectures and workshop were given by three faculty members who were experts on EBM and biostatistics.

### Intervention

A five-week EBM course was developed according to Kern’s six-step curriculum development approach [11]. The program has been designed around the basic steps of EBM [12].

Problem identification and general needs assessment: As the first step of program development, the World Federation of Medical Education’s Global Standards [13] and National Accreditation Standards [7] regarding teaching EBM have been taken into consideration.

Targeted needs assessment: The curriculum of CMF was evaluated. Although there were different lectures on EBM topics in some clerkship programs, there were not any EBM specific programs.

Aims and objectives: The aim of the program was to provide students with the basic knowledge and skills in the field of EBM and to enable students to understand the concept and application of EBM.

Educational Strategies: According to the Khan and Coomarasamy classification, the educational method of the course can be considered as Level IIa; Interactive and classroom based education [14]. We used homework assignments, interactive workshops, and critical appraisal sessions. The program was developed within the framework of pedagogical learning concepts, but it has also been influenced by adult learning theories [15, 16]. In accordance with adult learning theories, the course was offered as an extracurricular volunteer learning activity without any extrinsic motivational factors. Students signed up for the course by intrinsic motivation (humanistic theories); pre-test questions and test results allowed them to use critical reflection and helped them to be aware of knowledge gaps regarding EBM (transformative learning theory).

Course Program**:** The detailed course program and weekly schedules are explained in Table 1.

**Table 1:** Program Details

Week	Outline	Objectives	Content	Educational Method
		By the end of the session students will be able to:		
1.	▪ Pre-test ▪ Introduction to EBM practice ▪ Formulating focused clinical questions using the PICO formula	▪ Explain the concept of EBM ▪ List the components of the PICO formula ▪ Understand types of the PICO question ▪ Formulate a focused clinical question ▪ Value ​​using the most reliable scientific evidence as well as professional experience and patient’s values together on clinical decision	▪ Students were informed about the course ▪ Pre-test was applied before the main training started ▪ An interactive lecture was given about the concept of EBM and the creation of clinical questions using the PICO format ▪ A homework assignment was given at the end of the lecture	▪ Interactive classroom lecture ▪ Homework assignment
2	PubMed Class	▪ Explore different databases and understand their strengths and weaknesses ▪ Create search strategies by using Boolean operators (AND, OR, NOT) ▪ Perform MeSH database search ▪ Know how to find MeSH terms ▪ Use PubMed tags and filters	▪ The workshop was held in a computer lab where each student could work with an individual computer ▪ The training included the use of Boolean operators, MeSH database search, creating search strategies, using search filters, creating an NCBI account and setting e-mail alerts for saved searches	Workshop
3	Designing Scientific Research	▪ Name different types of scientific research designs ▪ Explain levels of evidence by using hierarchy of evidence pyramid ▪ Explain the concept of randomization ▪ Explain the importance of allocation concealment ▪ Understand the concept of blinding	The course content included introducing different study designs, randomized controlled trials, cohort studies, diagnostic and prognostic studies, bias in scientific research, prevention of bias, and the EBM pyramid.	Interactive classroom lecture
4	Biostatistics	▪ List the concepts used in the evaluation of statistical significance (such as *p* value, confidence interval, type I and type II errors) ▪ Give an example of confidence interval expressing statistical significance ▪ Calculate following measures given clinical scenarios o Sensitivity o Specificity o Positive predictive value o Negative predictive value o Probability ratio values o Absolute risk reduction o Relative risk reduction o Numbers Needed to Treat (NNT)	Statistic class was designed to understand the biostatistical approaches in scientific articles. Therefore, especially risk calculations, measures of diagnostic accuracy, Type I and II errors, confidence intervals, and *p* value were discussed.	Interactive classroom lecture
5	▪ Critical appraisal of scientific articles ▪ Post-test ▪ Students’ satisfaction survey	▪ Understand the concepts of internal and external validity of scientific research ▪ Explain the concept of bias ▪ List the most common types of bias ▪ Use the critical appraisal tools	▪ During the last week, the critical appraisal of scientific articles was discussed interactively ▪ The main theme of the training was the evaluation of the validity and reliability of scientific research using checklists ▪ After the completion of the fifth week of education, the post-test and the student satisfaction survey were applied	Critical appraisal workshop

**Fig. 1:**
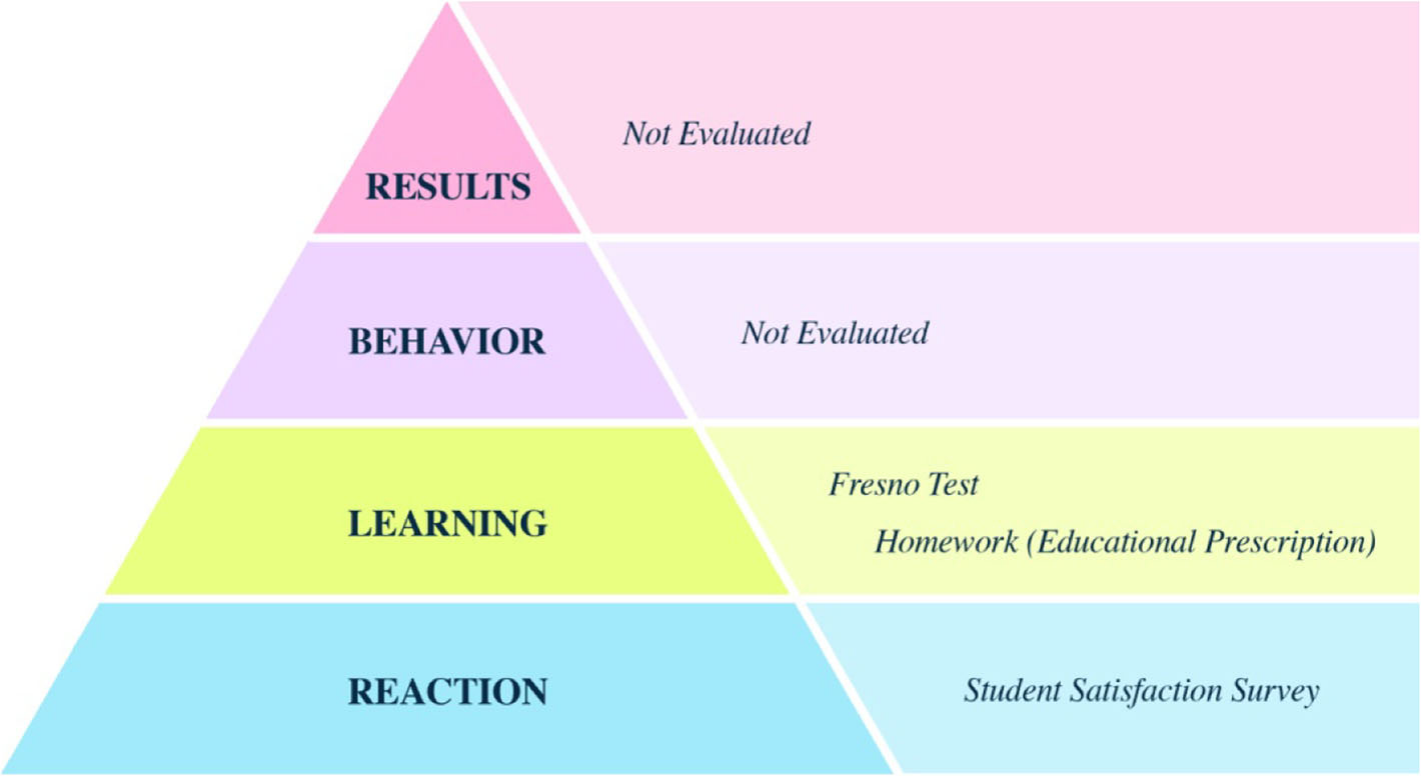
Evaluation steps and methods of the EBM course according to the Kirkpatrick Model

Evaluation: The first two steps of the Kirkpatrick Model for the program evaluation were used (Fig. 1) [17].

Kirkpatrick’s learning step and students’ success were assessed with the Turkish version of the Fresno Test [10]. The original Fresno Test was designed to assess the effectiveness of a comprehensive EBM curriculum at the University of California, San Francisco’s Fresno family practice residency program [18]. The test consists of 12 questions, starting with two clinical scenarios. Short answer questions about the clinical scenarios require the student to formulate a focused clinical question, identify the most appropriate research design to answer the question, show knowledge of database search methods, identify issues important for determining the relevance and validity of research articles, and discuss the magnitude and importance of research findings. The remaining questions, a series of statistical calculations and fill in the blank questions, are independent of these scenarios. The maximum score that can be achieved is 212. The test is scored by using a standardized system (Grading Rubric) [18]. There was a six-week time period between pre-test and post-test because of a one-week break between the fourth and fifth sessions. Both tests were identical.

Additionally, educational prescriptions were used as homework assignment. Students had to create a focused clinical question using the PICO format, acquire the best evidence, and appraise the evidence related to a specified clinical problem. Every student received feedback for homework.

Students’ satisfaction was assessed with a survey. All participants answered a survey that evaluated their satisfaction levels after the post-test application on the day they completed the training program. Evaluation of each weekly class as well as an overall program evaluation was asked for. Satisfaction scores ranged from 1 (not satisfied at all) to 10 (highly satisfied). Additionally, students were asked to report strengths, weaknesses, and opportunities of the program and how well it met their expectations. Also, the students’ opinion on which year of medical school would be most appropriate to teach the EBM program was asked.

Program Educators’ perspectives and thoughts were collected during regular review meetings and at the end of the course.

A SWOT (Strengths, Weaknesses, Opportunities, Threats) analysis was performed, using the data of the students’ survey, Fresno Test scores, and faculties’ opinions [19].

Timing of EBM Instruction: To determine which year of medical school is the most appropriate for the EBM program, following factors were taken into consideration: Comparison of students’ pre- and post-test scores between the third, fourth and fifth years of the medical school, students’ comments provided by survey, a curriculum analysis, and the number of applications from each class.

### Statistical analysis

The paired samples t-test and the McNemar test to compare pre-test and post-test scores as appropriate, and the Kruskal-Wallis test to compare students’ pre- and post-test scores between the third, fourth and fifth years of the medical school were used.

All data are reported as mean ± standard deviation (SD), or number (n) and percent (%).

Statistical significance was accepted at *p* < 0.05.

Statistical analysis was performed using STATA SE 14.2 (StataCorp LP, College Station, TX) and SPSS Statistics for Windows, Version 23.0. Armonk, NY: IBM Corp.

## Results

**Fig. 2:**
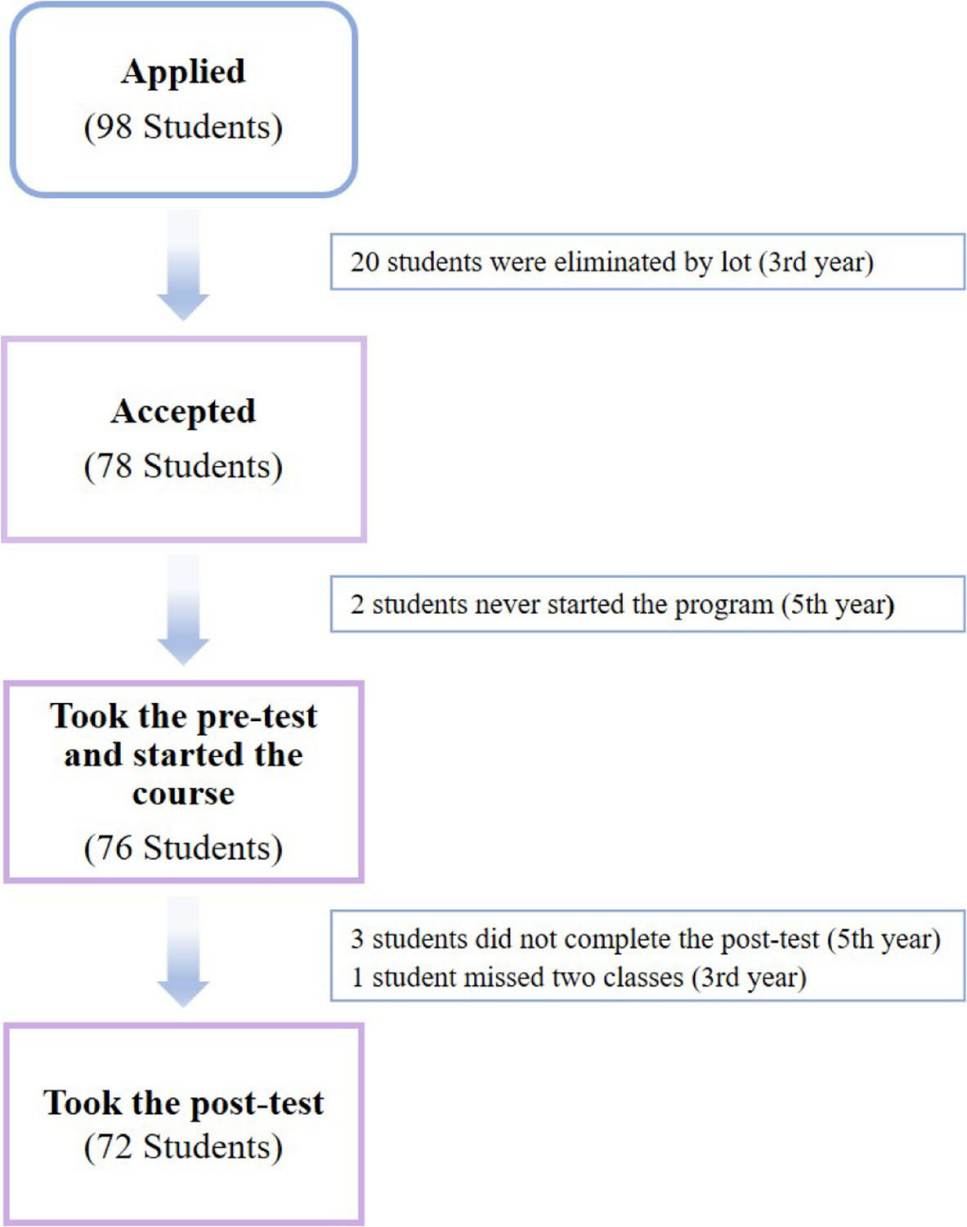
Flow-chart of the study participants

A total of 98 students applied to attend the program: 50 from the third, 19 from the fourth and 29 from the fifth year. Of the third-year students, 30 applications were randomly selected by lot. The number of applications from fourth year students was only 19, likely due to the high intensity of clinical clerkships during that year, and all were accepted. From the fifth year 29 students applied and were accepted. A total of 78 students were accepted and enrolled for the course. Two enrolled fifth year students never started the course. Among the remaining 76 students, four students were excluded from the analysis because three of them did not complete the post-test and one student did not attend two classes (Fig. 2).

Demographic data of the students are presented in Table 2.

**Table 2:** Demographic characteristics

	3rd Year	4th Year	5th Year	Total
Number of students	29	19	24	72
Program^b^				
Turkish	13 (34)	10 (26)	15 (40)	38
English	16 (47)	9 (26.5)	9 (26.5)	34
Age^a^	20,9 ± 1,04	21,8 ± 0,85	22,38 ± 0,49	21,6 ± 1,05
Gender^b^				
Female	15 (39)	11 (28)	13 (33)	39
Male	14 (42)	8 (25)	11 (33)	33

^a^Mean ± SD. ^b^Number (%)

### Students’ learning achievements

**Fig. 3:**
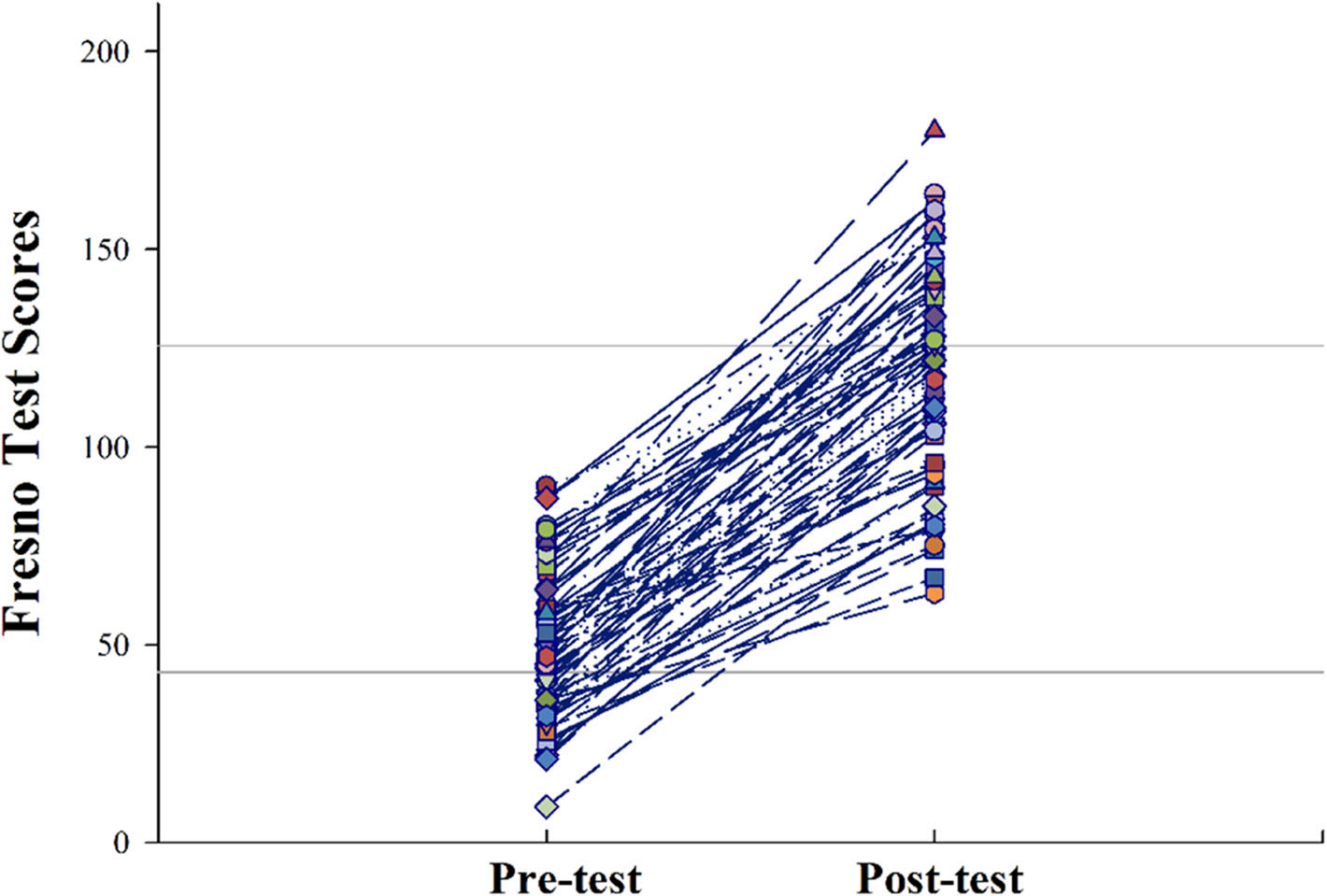
Mean pre- and post-test scores of all students (*n* = 72)

The average test score was 49.9 ± 18.2 out of 212 in the Turkish version of Fresno Test prior to participating in the course. The average post training score was 118.9 ± 26.3 with a change of 69 points (95% CI, 63.9–74.2) (Fig. 3). The Cohen’s effect size was 3.04 (95% CI, 2.6–3.5) indicating a very large change in scores.

Pre-test scores ranged from 9 to 90, and post-test scores from 63 to 180. The smallest individual increase in score from pre-test to post-test was 20, and the largest was 118. No student’s score declined from pre-test to post-test.

**Fig. 4:**
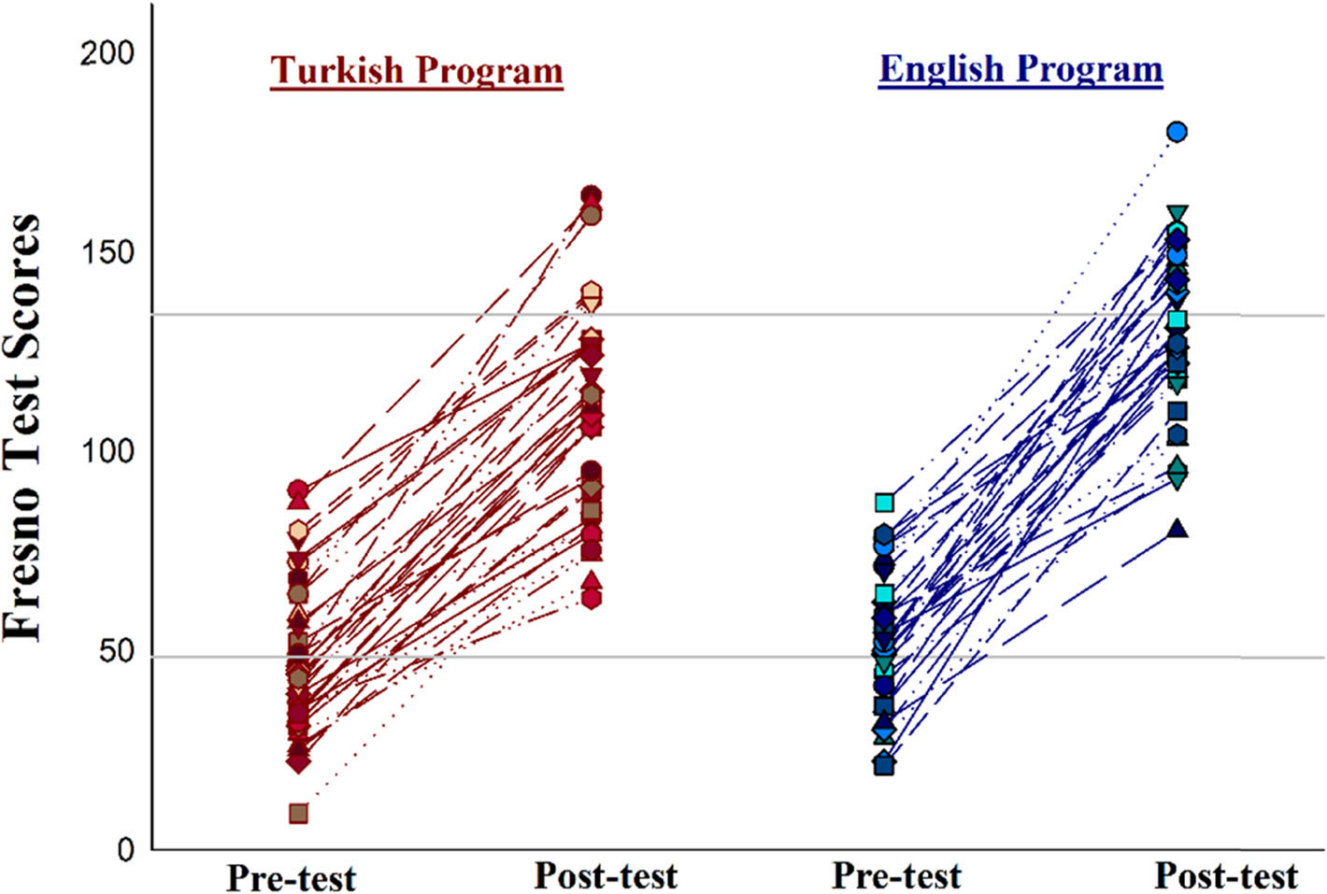
Mean pre- and post-test scores by program (Turkish Program *n* = 38, English Program *n* = 34)

A post-hoc comparison of test scores between Turkish and English Program students was performed (Table 3). Pre-test scores of Turkish Program students was 46.7 ± 19.1 and 53.2 ± 16.9 for English Program students (*p* = 0.154). Post-test scores of Turkish Program students (109.4 ± 26.4) were lower than those of the English Program (129.5 ± 21.9) (*p* = 0.001) (Fig. 4).

**Table 3:** Fresno Test scores of Turkish and English Program students

Program	Fresno Test scores^a^		Effect size^b^	*p*
	Pre-test	Post-test		
Turkish Program (n = 38)	46.7 ± 19.1	109.4. ±26.4	62.7 (55.7–69.2)	< 0.0001
English Program (n = 34)	53.2 ± 16.9	129.5. ±21.9	76.4 (69.22–83.54)	< 0.0001
*p*	0.54 (Pre-test comparison of two programs)	0.001 (Post-test comparison of two programs)	0.006 (Effect size comparison of two programs)	

^a^Mean ± SD, ^b^Effect size (CI %95)

When the Fresno Test results were analyzed on a question basis, there was a significant increase for all questions, except “diagnostic study design” question. We found the highest score increase for the “biomedical databases and developing search strategies” question.

### Students’ satisfaction

The overall satisfaction level of the students was 8.66 ± 1.09 out of 10. Students stated that the EBM course has met their expectations. No student commented negatively. The main aspects of the program from students’ perspective are summarized below.

#### Strengths of the program

- The program is structured to support scientific thinking rather than overloading the factual knowledge
- Increases the ability to follow scientific literature and to support continuous learning processes
- PubMed workshop was one of the strongest parts of the program
- Faculty members, who are the experts on the field of EBM
- Interactive teaching methods
- Getting prompt feedback on homework assignments
- Treats offered before and between classes
- Emailing system

#### Weaknesses of the program

- Short duration of the course
- Since the classes started after their daily school program students felt tired

#### Developmental aspects and recommendations for the program

- Extension of course duration
- Integrating with clinical practice
- Increasing the number of assignments
- Giving more time to the biostatistics course and making it more interactive
- Should stay as an elective course for allowing interactive teaching methods
- More articles should be included for critical appraisal

### Program evaluation

A SWOT analysis was performed for the strategic planning of future EBM courses (Table 4). For this analysis the Fresno Test scores and educational prescription homework results, the students’ satisfaction survey, and faculties’ observations and opinions were considered.

**Table 4:** SWOT Analysis

SWOT ANALYSIS	
**Strength**	**Weakness**
▪ Educational content of the program ▪ Students’ high level of knowledge acquisition ▪ Faculty members ▪ Encouraging critical thinking ▪ Providing guidance for lifelong learning	▪ Lack of educational material ▪ Short duration ▪ Class times (starts after daily schedule, so students feel tired)
**Opportunities**	**Threatens**
▪ Increasing importance given to evidence-based medical education ▪ The need for systematic training in EBM in CMF curriculum ▪ High interest of students for the program	▪ If the EBM course becomes compulsory, the interactive practice becomes more difficult due to an excess of class size

Timing of EBM Instruction: The Fresno Test scores did not differ among years of medical school (*p* = 0.37 for pre-test and *p* = 0.63 for post-test). Pre-test scores were 48.3 ± 17.2, 46.1 ± 16.6, and 54.8 ± 20.2 for the third, fourth and fifth year, respectively. Post-test scores were 122.1 ± 22.2, 115.5 ± 27.4, and 117.7 ± 30.3 in the same order.

Sixty-four percent of the students preferred the program to be offered during the second or third year of medical school. In order to preserve the interactive features of the education program, students suggested that the program should continue on a voluntary basis and be offered as elective course. The curriculum analysis showed that the EBM course could be integrated with the third year’s clinical biostatistics lectures. In addition, the application numbers from each year of medical school were considered. The highest number of applications (50 students) came from third year students, the lowest number (19 students) from fourth year students. Based on these results and with regards to the importance of early exposure to the EBM concept [20, 21], the course will be offered during the third year of medical school at CMF.

## Discussion

We newly developed an EBM training program at Istanbul University-Cerrahpaşa, CMF. The assessment of learning success using the Fresno Test shows effectiveness in improving students’ knowledge and skills in the field of EBM. To include this training as an elective course in the third year of the medical school was decided based on general evaluation results.

EBM can be thought in many different ways during the undergraduate medical education [3]. Despite the diversity of educational methods, the quantity and quality of the evidence to decide which is the most effective EBM teaching method is poor [4]. Systematic reviews have not identified a preferred method [22, 23]. Hatala and Guyatt stated that our knowledge about the outcomes of evidence-based curricula relies on observational data. Therefore, medical schools are recommended to develop local training programs according to the needs of their learners, program aims and objectives, and schools’ resources. Additionally, curricula should be developed around the main steps of EBM [12]. When we designed the EBM training program, we decided that the first three steps of EBM, “ask”, “search” and “appraise”, are best taught as a classroom-based EBM training. The other two steps, “application” and “evaluation”, are better addressed in clinical settings during bedside teaching [16]. Therefore, our course was designed to cover the first three steps of EBM. A longitudinal clinically integrated curriculum to cover the remaining steps of EBM is currently in planning.

Our EBM course is the first EBM program in Turkey that used a validated and internationally accepted EBM assessment tool, the Fresno Test [18]. This allowed us to compare the effectiveness of our course with other similar EBM programs in the world. The Fresno Test was first applied to family medicine residents and specialists during the development phase. While residents achieved an average of 95.5 points from the test, experts received 147.5 points [18]. In a multicenter study evaluating the evidence-based medical knowledge levels of medical school students in the United States, the mean score was found to be 102 for allopathic schools [24]. A study from Jordan showed that fifth-year medical students' pre-test scores were 26.7 out of 200 and 119.5 for the post-test after a 2-week short course. They slightly modified the scoring system of the Fresno Test from 212 to a 200 total score [25]. In a Spanish study, residents’ Fresno Test scores increased from 63.9 to 111.6 after an EBM course [26]. Our students have not been exposed to any structured EBM training before our newly developed program which explains their low pre-test scores. The improvement of Fresno Test scores is relevant and consistent with the other international studies [25–27].

On detailed review of pre- and post-test scores for each question, we identified areas of limited improvement of student scores. This was especially the case for biostatics topics. This information allowed us to refine the course. For future courses, we decided to allocate more time for statistical lectures, and to add interactive sessions that allow students to perform more calculations and interpretations of statistical analyses.

Although it was not our hypothesis, we found that there was a significant difference between Turkish and English Program students’ post-test scores. While pre-test scores were comparable, English Program students achieved higher post-test scores. We cannot explain this interesting observation with our study data. But we aim to follow up with future studies.

We suggest including EBM training as an elective course in the third year of the medical school for several reasons. The high number of course applications from third year students (50 applications) was a strong indicator of interest. In addition, we considered the results of the student satisfaction surveys and faculty evaluation. Moreover, we believe that the early introduction of EBM provides benefits. Although there is no strong evidence on the most effective time to start EBM training [3], a pre-clinical implementation might provide educators an extended timeframe to teach longitudinal programs rather than a single innovation. Additionally, early exposure to EBM provides an increase of the students’ self-efficacy in their ability to practice EBM [20]. Previous research also suggested that the introduction of EBM in preclinical years increases the students’ understanding of the conduct of medical research and that EBM is a necessary foundation to develop clinical skills [21].

This study has limitations. The EBM course was an extra-curricular activity that was carried out over a limited time. Student achievements, early knowledge and skills acquisitions are evaluated at the end of the program. Long-term results of education such as behavioral change or effect on health care were not investigated in the study. Therefore, the sixth step of Kern’s framework for curriculum development (Evaluating the effectiveness of the curriculum) was not completed. In addition, the program was designed to cover the first three steps of EBM only. We are planning to integrate the fourth and fifth step with clinical bedside teaching. However, this was out of the scope of the current study. In addition, the timing of the course after students’ usual daily program might have affected students’ ability to concentrate and learn. We did not use the exam scores for pass or fail decisions, and the results did not affect students’ medical school scores. The training was separate from their schoolwork. Therefore, the exam scores obtained with the Fresno Test were only depended on the students’ interest and their self-direction without any external motivation. If this course was mandatory, results might have been different.

## Conclusions

Evidence-based practice is one of the essential elements for improving the quality of health services, reducing healthcare costs and preventing medical errors [28]. One of the responsibilities of medical schools is to graduate physicians who have reached the necessary qualifications in this field by including EBM programs in their curriculums. CMF’s EBM program can serve as an example of an effective training for medical schools that have not yet included EBM education into their curriculum. The reported Fresno Test results might allow to compare the students’ achievements with other national and international programs.

## Data Availability

The datasets analyzed during the current study are available from the corresponding author on reasonable request.
